# The association between physical fitness and depressive symptoms among young adults: results of the Northern Finland 1966 birth cohort study

**DOI:** 10.1186/1471-2458-13-535

**Published:** 2013-06-03

**Authors:** Kadri Suija, Markku Timonen, Maarit Suviola, Jari Jokelainen, Marjo-Riitta Järvelin, Tuija Tammelin

**Affiliations:** 1 Institute of Health Sciences, University of Oulu, Box 5000, FIN 90014, Oulu, Finland; 2 Department of Family Medicine, University of Tartu, Puusepa 1a, 50406, Tartu, Estonia; 3 Unit of General Practice, Oulu University Hospital, Oulu, FIN, 90029, Finland; 4 National Public Health Institute, P.O. Box 310, Oulu, FIN, 90101, Finland; 5 Imperial College, London, UK; 6 LIKES – Research Center for Sport and Health Sciences, Viitaniementie 15a, Jyväskylä, FIN, 40720, Finland; 7 Finnish Institute of Occupational Health, Aapistie 1, Oulu, FIN, 90220, Finland

**Keywords:** Physical fitness, Physical activity, Cardiorespiratory fitness, Muscular fitness, Depressive symptoms, Depression, Young adults

## Abstract

**Background:**

The effect of physical activity on mental health has been the subject of research for several decades. However, there is a lack of studies investigating the association between physical fitness, including both cardiorespiratory and muscular fitness and depressive symptoms among general population. The aim of this study was to determine the association between physical fitness and depressive symptoms among young adults.

**Methods:**

The study population consists of 5497 males and females, members of the Northern Finland birth cohort of 1966, who at age 31 completed fitness tests and filled in a questionnaire including questions about depressive symptoms (Hopkins’ Symptom Checklist-25) and physical activity. Cardiorespiratory fitness was measured by a 4-min step test and muscular fitness by tests of maximal isometric handgrip and isometric trunk extension. The odds ratios (OR) with 95% confidence intervals (95% CI) for having depressive symptoms were calculated for quintiles groups of physical fitness using the third, median quintile as reference group, and the results were adjusted for potential confounding variables.

**Results:**

Depressive symptoms were most common among males and females in the lowest quintile group of trunk extension test (OR 1.58 and 95% CI 1.07-2.32 in males and OR 1.43 and 95% CI 1.03-2.0 in females) and among males in the lowest quintile group of handgrip strength (OR 1.64 95% CI 1.11-2.42) compared to the reference group. Level of self-reported physical activity was inversely associated with depressive symptoms both in males (OR 1.74 95% CI 1.25-2.36) and females (OR 1.36 95% CI 1.05-1.75). The cardiorespiratory fitness was not associated with depressive symptoms (OR 1.01 95% CI 0.68-1.49 in males and 0.82 95% CI 0.57-1.16 in females).

**Conclusions:**

The results indicate that low level of isometric endurance capacity of trunk extensor muscles is associated with high level of depressive symptoms in both sexes. In males, also poor handgrip strength is associated with increased levels of depressive symptoms. The physical activity level is inversely associated with the prevalence of depressive symptoms among young adults.

## Background

The effect of physical activity on mental health has been the subject of research for several decades. Epidemiological studies of community samples have demonstrated that greater amounts of physical activity are generally associated with reduced depressive symptoms [1-4]. In addition, the large population-based study has also presented an inverse graded dose–response relationship between maximal cardiorespiratory fitness and depressive symptoms [2]. There are also studies about depressive symptoms and other physical fitness measurements. For example the Netherlands Study of Depression and Anxiety showed that women with depressive disorder had lower handgrip strength in comparison with healthy women [5]. Similarly some aging studies have found negative associations between handgrip strength and depression [6]. Further, individuals who are physically more fit, are also less likely to be concurrently diagnosed as having clinical depression [7-10].

The interpretation of the results of different earlier studies concerning the association between physical fitness/activity and depressive symptoms is hampered because of several methodological discrepancies in the study designs and case definitions for both depression or depressive symptoms and physical activity. To the best of our knowledge, epidemiological studies examining physical activity and depressive symptoms have used questionnaires and structured interviews for measuring physical activity and depressive symptoms. None of the large epidemiological studies have investigated the association between physical fitness including both cardiorespiratory and muscular fitness and depressive symptoms in young adults. Objective measurements of physical fitness would provide complimentary information to self-reported physical activity and fitness [11].

The aims of the current study were to determine the cross-sectional associations between: 1) physical activity and depressive symptoms, and 2) physical fitness, including both cardiorespiratory and muscular fitness and depressive symptoms among young adults. We suggested that low levels of physical fitness and physical activity are associated with higher prevalence of depressive symptoms among young adults.

## Methods

### Study population

The study population consisted of the Northern Finland birth cohort of 1966, originally including all 12 231 males and females whose expected year of birth was 1966 [12]. In 1997–1998, at age 31, all the members of the cohort who were still alive (N = 11 541) were invited to participate in a follow-up survey. A questionnaire including questions about depressive symptoms and physical activity was mailed to this group, and 8767 (76%) persons responded. The subjects who were living in the Northern Finland, or had moved to the Helsinki capital area, were also invited to a medical examination performed in local health centres. The invitation to participate in the medical examination was accepted by 6033 persons (71% of those invited), and 5497 (65% of those invited) males and females completed all fitness tests. Altogether 51% of them were women, 73% were married or cohabiting and 71% were employed.

The Ethics Committee of Oulu University Hospital has approved this study. Written informed consent was obtained from all the subjects before participation in the study.

### Variables

#### Depressive symptoms

Information on depressive symptoms was obtained through Hopkins’ Symptom Checklist-25 (HSCL-25) [13-17] which was included in the above-mentioned postal questionnaires. HSCL-25 is a 25-item shortened version of an originally 90-item questionnaire designed by Derogatis et al. [13]. A depression subscale consists of 13 items [13,14,17]. Cohort members recorded their estimates of severity of their depressive symptoms on a scale ranging from 1 (“not at all”) to 4 (“extremely”). Responses were summed and divided by the number of answered items to generate a depressive symptoms mean score ranging from 1.0 to 4.0. There are two commonly used mean scores of 1.55 and 1.75 [13,14,17]. These points are cut off points for important depressive symptoms but not for diagnosis of major depression.

#### Physical fitness

Four teams of trained research nurses, who supervised the fitness tests and performed the anthropometric measurements, carried out the medical examinations. Before the fitness test, the subjects were interviewed to screen out persons with cardiovascular diseases or orthopaedic problems. Less than 10% of all subjects were excluded from the analyses for various reasons. The most common reasons for not performing step or trunk extension tests at this age of 31 years were ill health and pregnancy [18].

#### Cardiorespiratory fitness

In the medical examination, the subjects performed a submaximal four-minute single step test conducted without shoes on a bench 33 cm high for the females and 40 cm high for the males [19]. We used heart rate after step test as an indicator of cardiorespiratory fitness. A metronome paced a rate of 23 steps per minute. Heart rate (beats min^-1^) was measured immediately after the test by a heart rate monitor handle on the chest (Fitwatch, Polar Electro, Kempele, Finland). In a laboratory measurements of 123 adults aged 31 years the correlation coefficient between heart rate after step test and peak oxygen uptake during maximal exercise test was 0.53 [19].

#### Muscular fitness

Muscular fitness was measured by trunk extension test and maximal isometric handgrip test. During the *trunk extension test*[18,20] the subject was in a prone position, the lower body lying on the stand and the upper body unsupported from the level of the anterior superior iliac spine upwards. The tester sitting on them stabilized the legs and the arms were held beside the trunk. The isometric endurance capacity of the trunk extensor muscles was evaluated by holding the upper part of the body in a horizontal position as long as possible, however, not exceeding four minutes. When the subject was no longer able to maintain the horizontal position, the test ended. The outcome measure of the test was the endurance time in seconds.

*Maximal isometric handgrip strength* of the dominant hand was measured with a hand dynamometer (Newtest, Oulu, Finland) based on the strain-gauge technique. Measurements were performed with the subject in a standing position, holding the dynamometer, with the hand beside but not touching the trunk. The wrist and the elbow were extended. The width of the grip in the dynamometer was adjusted to the size of the hand. The highest value in Newton (N) of the three trials, each lasting from two to four seconds, was accepted as the result [18].

#### Leisure-time physical activity

In the above-mentioned postal questionnaires, subjects were also asked how often they participated in light and brisk physical activities. Response alternatives in this study were daily, four to six times a week, two to three times a week, once a week, two to three times a month, and once a month or less often. The duration of one bout of activity was considered separately for light and brisk activities with the following alternatives: more than 90 minutes, 60–90 minutes, 40–59 minutes, 20–39 minutes, less than 20 minutes, and not at all. In the questionnaire, the term ‘brisk’ was defined as physical activity causing at least some sweating and getting out of breath, and the term ‘light’ as physical activity causing no sweating or getting out of breath. Total volume of leisure-time physical activity was expressed as metabolic equivalent hours/week (MET-hours/week), which was formed by calculating duration and frequency of both brisk and light physical activity. In the calculations, an intensity value of 3 METs was used for light physical activity and 5 METs for brisk physical activity. We formed five equally distributed categories to describe quintiles of physical activity (Q1-Q5) (Table 1).

**Table 1:** Prevalence of depressive symptoms according to quintiles groups of cardiorespiratory fitness, muscular fitness and physical activity

**Male**		**Female**	
**HSCL-25 - 1.75**		**HSCL-25 - 1.75**	
**% (95% CI)**		**% (95% CI)**	
**Cardiorespiratory fitness, heart rate after step test, beats/min**			
Q1 (161–197)	11.4 (8.9-14.4)	Q1 (166–199)	13.2 (10.5-16.4)
Q2 (152–160)	8.5 (6.3-11.2)	Q2 (157–165)	15.4 (12.6-18.7)
Q3 (143–151)	10.6 (8.4-13.4)	Q3 (148–156)	15.6 (12.9-18.8)
Q4 (134–142)	11.3 (8.8-14.4)	Q4 (137–147)	17.1 (14.2-20.5)
Q5 (76–133)	7.8 (5.9-10.4)	Q5 (74–136)	17.9 (15.0-21.3)
	P = 0.163^*^		P = 0.255^*^
**Muscular fitness, trunk extension test, s**			
Q1 (1–120)	14.8 (12.1-18.0)	Q1 (10–125)	20.7 (17.6-24.2)
Q2 (121–147)	11.3 (8.9-14.3)	Q2 (126–165)	17.3 (14.3-20.7)
Q3 (148–177)	10.3 (8.0-13.2)	Q3 (166–211)	14.6 (11.9-17.8)
Q4 (178–214)	6.7 (4.9-9.1)	Q4 (212–240)	13.8 (11.9-16.0)
Q5 (215–240)	7.5 (5.5-10.0)		
	P < 0.001^*^		P = 0.002^*^
**Muscular fitness, handgrip test, kg**			
Q1 (15.8-42.4)	14.4 (11.7-17.6)	Q1 (3.3-23.6)	15.1 (12.5-18.2)
Q2 (42.5-47.5)	10.9 (8.6-13.8)	Q2 (23.7-26.6)	15.2 (12.5-18.3)
Q3 (47.6-51.7)	9.4 (7.2-12.1)	Q3 (26.7-29.3)	16.7 (13.9-19.9)
Q4 (51.8-56.3)	8.6 (6.5-11.3)	Q4 (29.4-32.3)	15.7 (13.0-18.9)
Q5 (56.4-99.0)	8.0 (6.0-10.6)	Q5 (32.4-90.5)	18.5 (15.6-21.8)
	P = 0.004^*^		P = 0.485^*^
**Physical activity, MET-hours/week**			
Q1 (≤1.6)	17.9 (15.4-20.7)	Q1 (≤3.7)	21.3 (18.8-24.0)
Q2 (1.7-7.4)	11.3 (9.3-13.8)	Q2 (3.8-9.4)	18.9 (16.4-21.6)
Q3 (7.5-13.7)	9.6 (7.8-11.8)	Q3 (9.5-15.5)	15.7 (13.4-18.3)
Q4 (13.8-26.1)	9.5 (7.7-11.8)	Q4 (15.6-26.1)	15.0 (12.8-17.4)
Q5 (26.2-84.0)	8.8 (7.0-11.0)	Q5 (26.2-84.0)	15.7 (13.3-18.3)
	P < 0.001^*^		P = 0.001^*^

HSCL-25 = Hopkins’ Symptom Checklist-25, cut-off point 1.75.

95% CI = 95% confidence interval.

MET = metabolic equivalent.

Q = quintile of corresponding physical activity or fitness variable.

^*^Chi-Square-test p-values.

#### Potential confounding variables

Since alcohol intake, obesity, smoking and somatic diseases have been shown to be associated with depressive symptoms [21-23] and physical fitness [24,25], those were used as potential confounding factors in multivariate regression analyses.

#### Alcohol intake

Information on the frequencies of beer, wine and other spirit consumptions as well as statements on the usual amounts of each alcoholic drink per one drinking occasion was requested in the questionnaire. For each type of drink (the alcohol percentages of which were turned into amounts of pure alcohol consumed), the frequency of alcohol use was proportioned to 365 days. The average amount of pure alcohol (g/day) was calculated as follows: pure alcohol (g) at any one time x frequency of alcohol use (1/day). Alcohol consumption was categorized as abstainers/light drinkers (< 15 g of pure alcohol/day), moderate drinkers (15–40 g/day), and heavy drinkers (> 40 g/day) [26].

#### Obesity

Body mass index (BMI) was calculated as weight/height^2^ (kg.m^-2^). Body height and weight were measured to an accuracy of 0.1 cm and 0.1 kg, respectively.

#### Smoking

Cohort member’s regular daily smoking at the age of 31 was classified as follows: regular smokers (i.e. smoking on 7 days a week), occasional smokers, (i.e. smoking less than on 7 days per week) and non-smokers [27].

#### Somatic diseases

We used data of national Finnish hospital discharge register about the presence of lifetime hospital-treated somatic disease [28].

#### Statistical methods

The prevalence of depressive symptoms (as defined by the HSCL-25 depression subscale mean score cut off points of 1.55 and 1.75) was compared in quintiles groups of physical activity and fitness (Q1-Q5). In order to adjust the results for potential confounding variables, multiple binary logistic regression analyses were used. Odds ratios (OR) with 95% confidence intervals (95% CI) were calculated for having depressive symptoms by different quintiles of physical activity and fitness. The third quintile group (Q3) was used as reference group.

All statistical analyses were performed using the statistical program Stata (Stata Statistical Software: Release 11. StataCorp. 2009. College Station, TX: StataCorp LP.).

## Results

The prevalence of depressive symptoms as defined by the HSCL-25 depression subscale mean score of 1.55 and 1.75 in males was 18.2% and 11.7%, respectively. The corresponding numbers for females were 26.0% and 17.4%. The results were similar (no significant differences) by using different cut-off values (1.55 or 1.75) and therefore only results for cut-off score 1.75 are presented in tables.

The prevalence of depressive symptoms in quintile groups of physical activity and fitness are presented in Table 1. The prevalence of depressive symptoms increased in line with decreasing result of trunk extension test in both genders. For example, 14.8% of males and 20.7% of females in the lowest quintile and 7.5% in the highest quintile had depressive symptoms. Results of the handgrip test were related with depressive symptoms in males (p = 0.004) but not in females (p = 0.485). The physical activity level (MET-hours/week) was inversely associated with the prevalence of depressive symptoms in both genders, but cardiorespiratory fitness was not associated with depressive symptoms in either males or females.

The results after adjusting for potential confounding factors (alcohol intake, smoking, obesity, somatic diseases) are presented in Table 2. The third quintile (Q3) of each variable was used as the reference group (ref). Regarding the trunk extension test, the ORs of depressive symptoms in the lowest quintile was 1.58 (95% CI, 1.07-2.32) for males and 1.43 (95% CI, 1.03-2.0) for females. Concerning the handgrip test, the ORs of depressive symptoms in the lowest quintile was 1.64 (95% CI, 1.11-2.42) in males and 0.86 (95% CI, 0.63-1.18) in females.

**Table 2:** Multivariate logistic regression analysis of depressive symptoms using Hopkins’ Symptom Checklist-25

	**Depressive symptoms (HSCL-25 - 1.75)**			
	**Male**		**Female**	
	**Crude**	Adjusted^*^	**Crude**	Adjusted^*^
	**OR (95% CI)**	**OR (95% CI)**	**OR (95% CI)**	**OR (95% CI)**
**Cardiorespiratory fitness, heart rate after step test, beats/min**				
Q1	1.08 (0.74-1.57)	1.01 (0.68-1.49)	0.82 (0.59-1.15)	0.82 (0.57-1.16)
Q2	0.78 (0.52-1.17)	0.8 (0.53-1.21)	0.99 (0.72-1.36)	1.01 (0.72-1.4)
Q3	ref.	ref.	ref.	ref.
Q4	1.07 (0.73-1.57)	1.05 (0.71-1.56)	1.12 (0.82-1.53)	1.18 (0.85-1.63)
Q5	0.72 (0.48-1.07)	0.65 (0.42-1.0)	1.18 (0.87-1.6)	1.24 (0.9-1.71)
**Muscular fitness, trunk extension test, s**				
Q1	1.52 (1.05-2.18)	1.58 (1.07-2.32)	1.53 (1.12-2.08)	1.43 (1.03-2.0)
Q2	1.11 (0.75-1.64)	1.07 (0.71-1.61)	1.22 (0.88-1.69)	1.13 (0.81-1.58)
Q3	ref.	ref.	ref.	ref.
Q4	0.62 (0.4-0.97)	0.66 (0.42-1.04)	0.94 (0.7-1.25)	0.96 (0.71-1.29)
Q5	0.7 (0.46-1.08)	0.74 (0.48-1.16)	(-)	(-)
**Muscular fitness, handgrip test, kg**				
Q1	1.63 (1.12-2.36)	1.64 (1.11-2.42)	0.89 (0.65-1.21)	0.86 (0.63-1.18)
Q2	1.18 (0.8-1.75)	1.25 (0.83-1.87)	0.89 (0.66-1.22)	0.87 (0.63-1.19)
Q3	ref.	ref.	ref.	ref.
Q4	0.91 (0.6-1.38)	1 (0.66-1.54)	0.93 (0.68-1.26)	0.88 (0.64-1.21)
Q5	0.84 (0.55-1.29)	0.87 (0.56-1.35)	1.13 (0.84-1.52)	1.08 (0.8-1.47)
**Physical activity, MET-hours/week**				
Q1	2.05 (1.53-2.75)	1.74 (1.29-2.36)	1.45 (1.14-1.85)	1.36 (1.05-1.75)
Q2	1.2 (0.87-1.65)	1.1 (0.79-1.53)	1.25 (0.97-1.61)	1.3 (1.0-1.7)
Q3	ref.	ref.	ref.	ref.
Q4	0.99 (0.71-1.38)	0.96 (0.68-1.35)	0.94 (0.73-1.22)	0.99 (0.76-1.29)
Q5	0.91 (0.65-1.27)	0.92 (0.65-1.31)	1 (0.77-1.3)	1.05 (0.8-1.38)

HSCL-25 = Hopkins’ Symptom Checklist-25, cut-off point 1.75.

OR = Odds ratio.

95% CI = 95% confidence interval.

MET = metabolic equivalent.

Q = quintile of corresponding physical activity or fitness variable.

^*^adjusted to potential confounding variables (alcohol intake, smoking, obesity, somatic diseases).

The lowest quintile of physical activity (≤1.6 MET-hours/week) associated with increased likelihood of depressive symptoms in males: OR was 1.74 (95% CI, 1.25-2.36). In females, the two lowest quintiles (≤9.4 MET-hours/week) associated with depressive symptoms as defined by cut-off point of 1.75 ORs being up to 1.36 (95% CI, 1.05-1.75).

With regard to cardiorespiratory fitness, the highest quintile associated with decreased level of depressive symptoms (OR = 0.65, 95% CI 0.42-1.0) in males using the HSCL-25 cut-off point of 1.75.

## Discussion

Our study shows that high level of isometric endurance capacity of trunk extensor muscles is associated with low levels of depressive symptoms in both males and females. In males, also poor handgrip strength was associated with increased levels of depressive symptoms. Our results are consistent with previous research showing the association between handgrip strength and depressive symptoms [5,6]. However, these earlier studies have been conducted among middle-aged or elderly population. Therefore, the present study provides new information about the inverse association between muscular fitness and depressive symptoms also among young adults.

We also found that the low level of self-reported physical activity was significantly associated with increased prevalence of depressive symptoms among young adults. The association between depressive symptoms and low level of self-reported physical activity has already been shown in several previous studies [1-3].

Interestingly, there was no significant association between objectively measured cardiorespiratory fitness and depressive symptoms in either males or females. These results are not consistent with the evidence from the first community-based observational study demonstrating the inverse association between maximal cardiorespiratory fitness and depressive symptoms [2] in which participants completed a maximal exercise treadmill test to estimate cardiorespiratory fitness. However, in our study, we used submaximal step test to measure cardiorespiratory fitness. The main reason we performed submaximal exercise test was that direct measurement of maximum oxygen consumption (VO2max) during a maximal exercise test is time-consuming, requires laboratory equipment and involves health risks especially in large populations. The use of different measurement methods (submaximal vs. maximal exercise test) may explain the differences in the results regarding cardiorespiratory fitness.

On the other hand, in our study, muscular fitness was measured by maximal trunk extension test and maximal handgrip test. It may be that the results from physical fitness tests that need maximal physical effort and at the same time maximal mental motivation to perform the test maximally are more strongly associated with depressive symptoms. This maximal or submaximal physical effort needed during the certain fitness test may explain why results of the submaximal step test were not associated with depressive symptoms.

Major depressive disorder is a highly prevalent condition that worsens functioning and quality of life [29]. Furthermore, the number of people diagnosed as having depressive disorder is rising [30]. The identification of prevention and invention strategies aimed at decreasing depressive symptom, which can be applied to populations inexpensively and without side effects, would be needed. In clinical practice, physical activity and subsequent good physical fitness inevitably have potential benefits with very few obvious risks. Physical activity is a particularly beneficial behaviour due to the combined effects on both physical and mental health [1,9]. Several mechanisms, how exercise affects depressive symptoms, have been proposed. We speculate that physical inactivity is likely to be both a cause and consequence of depressive symptoms. As depression can be characterized by low energy, it is possible that depression leads to decreased physical activity and physical fitness. People who are depressed may be less likely to engage in physical activities [31,32] and, on the other hand, good mental health increases likelihood of engaging in physical activity [33]. However, there are also studies showing no effect of exercise on depressive symptoms [34]. According to the recent systematic review, the effectiveness of exercise on treatment of major depression is still unknown [35]. Even causality between depressive symptoms and physical activity/fitness could not be evaluated; our study presents associations with several different measures of physical fitness.

### Study limitations and strengths

Limitations of our study are the cross-sectional nature of these data that does not allow inferences about causality. Further, the HSCL-25 does not provide a specific depression diagnosis like structured clinical interviews. The weakness of the study is also that we used self-reported leisure-time physical activity. In addition, cardiorespiratory fitness was measured by using the submaximal step test and not by a maximal exercise test, which is regarded more accurate test to evaluate cardiorespiratory fitness.

The strengths of our study were that it was based on a large representative sample (N = 5497) of young (31 years) people born in the North of Finland. Extensive data based on the general population, describing people of different socioeconomic classes, offers a unique opportunity to study the association between depressive symptoms and physical fitness. Additionally, HSCL-25 has proved to be an acceptable screening scale for obtaining information on symptoms of depression among large population [15].

Even we used self-reported questionnaire to determine physical activity, objective measurements of muscular fitness by maximal tests can be considered strength of this study.

## Conclusions

Our novel finding was that a low level of isometric endurance capacity of trunk extensor muscles is associated with high level of depressive symptoms in both sexes. In males, also poor handgrip strength was associated with increased levels of depressive symptoms. The physical activity level was inversely associated with the prevalence of depressive symptoms among young adults. Further investigations are needed to clarify gender differences and whether it is specifically the maximal effort needed in physical fitness test that explains the observed association between poor results in maximal fitness test and depressive symptoms.

## Abbreviations

OR: Odds Ratio; 95% CI: 95% Confidence interval; MET: Metabolic equivalent; N: Newton; BMI: Body mass index; HSCL-25: Hopkins’ Symptom Checklist-2; ref: Reference group; V02max: Maximum oxygen consumption.

## Competing interest

The authors declare that they have no competing interests.

## Authors’ contributions

KS interpreted the results and drafted the manuscript. MT participated in the design of the study and investigation. MS analysed the data and helped to draft the manuscript. JJ performed the statistical analysis. MRJ participated in the design of the study. TT conceived of the study and participated in its design. All authors participated in the write-up, contributed to the interpretation of the study results and approved the final version of the manuscript submitted for publication. All authors read and approved the final manuscript.

## Pre-publication history

The pre-publication history for this paper can be accessed here:

http://www.biomedcentral.com/1471-2458/13/535/prepub
